# Alternariol

**DOI:** 10.1107/S1600536810017502

**Published:** 2010-05-15

**Authors:** David Siegel, Sergey Troyanov, Johannes Noack, Franziska Emmerling, Irene Nehls

**Affiliations:** a Bundesanstalt fur Materialforschung und -prüfung, Abteilung Analytische Chemie; Referenzmaterialien, Richard-Willstätter-Strasse 11, D-12489 Berlin-Adlershof, Germany; b Humboldt-Universität zu Berlin, Department of Chemistry, Brook-Taylor-Strasse 2, 12489 Berlin, Germany

## Abstract

In the title compound (systematic name: 3,7,9-trihydr­oxy-1-methyl-6*H*-benzo[*c*]chromen-6-one), C_14_H_10_O_5_, the methyl group is shifted out of the molecular plane due to a steric collision, thus causing a slight twist of the benzene rings. The mol­ecular structure is stabilized by an intra­molecular O—H⋯O hydrogen bond, generating an *S*(6) ring. In the crystal, mol­ecules are connected by inter­molecular O—H⋯O hydrogen bonds into a three-dimensional network.

## Related literature

Alternariol is a mycotoxin (toxic secondary fungal metabolite) produced by ubiquitous *Alternaria* moulds. For information on occurence and toxicity, see: Weidenbörner (2001 ▶); Brugger *et al.* (2006 ▶); Wollenhaupt *et al.* (2008 ▶); Fehr *et al.* (2009 ▶). For crystallization, alternariol was obtained by total synthesis according to Koch *et al.* (2005 ▶). For a comparable structure, (2-chloro-7-hydr­oxy-8-methyl-6*H*-benzo[*c*]chromen-6-one), see: Appel *et al.* (2006 ▶).
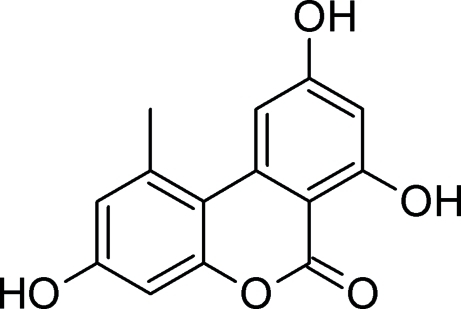


## Experimental

### 

#### Crystal data

C_14_H_10_O_5_
*M* *_r_* = 258.22
Orthorhombic, 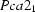

*a* = 18.969 (3) Å
*b* = 3.7244 (6) Å
*c* = 15.235 (3) Å
*V* = 1076.3 (3) Å^3^
*Z* = 4
Mo *K*α radiation
μ = 0.12 mm^−1^
*T* = 150 K
0.40 × 0.10 × 0.02 mm

#### Data collection

Stoe IPDS diffractometer
6072 measured reflections
1338 independent reflections
1053 reflections with *I* > 2σ(*I*)
*R*_int_ = 0.067

#### Refinement

*R*[*F* ^2^ > 2σ(*F* ^2^)] = 0.035
*wR*(*F* ^2^) = 0.069
*S* = 0.99
1338 reflections
191 parameters
1 restraint
H atoms treated by a mixture of independent and constrained refinement
Δρ_max_ = 0.16 e Å^−3^
Δρ_min_ = −0.23 e Å^−3^

### 

Data collection: *X-AREA* (Stoe & Cie, 2006 ▶); cell refinement: *X-AREA*; data reduction: *X-AREA*; program(s) used to solve structure: *SHELXS97* (Sheldrick, 2008 ▶); program(s) used to refine structure: *SHELXL97* (Sheldrick, 2008 ▶); molecular graphics: *DIAMOND* (Brandenburg, 2010 ▶) and *ORTEPIII* (Burnett & Johnson, 1996 ▶); software used to prepare material for publication: *SHELXTL* (Sheldrick, 2008 ▶).

## Supplementary Material

Crystal structure: contains datablocks I, global. DOI: 10.1107/S1600536810017502/bt5266sup1.cif

Structure factors: contains datablocks I. DOI: 10.1107/S1600536810017502/bt5266Isup2.hkl

Additional supplementary materials:  crystallographic information; 3D view; checkCIF report

## Figures and Tables

**Table 1:** Hydrogen-bond geometry (Å, °)

*D*—H⋯*A*	*D*—H	H⋯*A*	*D*⋯*A*	*D*—H⋯*A*
O3—H3⋯O2	0.86 (3)	1.82 (3)	2.605 (3)	152 (3)
O4—H4⋯O2^i^	0.91 (3)	1.81 (3)	2.685 (3)	162 (3)
O5—H5⋯O4^ii^	0.84	1.97	2.809 (2)	175

Symmetry codes: (i) 
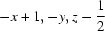
; (ii) 
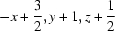
.

**Fig. 1.**
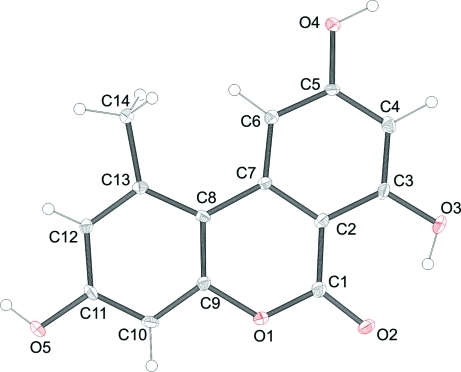
: ORTEP representation of the title compound with atomic labeling shown with 30% probability displacement ellipsoids.

**Fig. 2.**
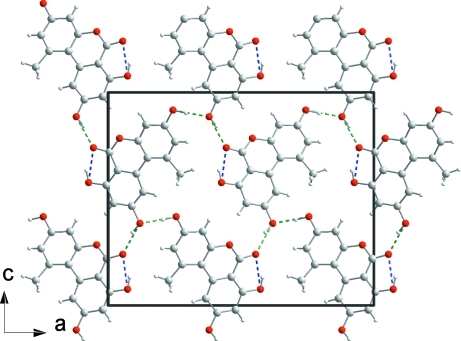
: View of the unit cell of the title compound along [010], showing the hydrogen-bonded layers.

## supplementary crystallographic information

### Comment

Alternariol is a cytotoxic, fetotoxic, teratogenic (Weidenbörner, 2001), mutagenic (Brugger *et al.*, 2006, Wollenhaupt *et al.*, 2008) and genotoxic (Fehr *et al.*, 2009) mycotoxin produced by ubiquitous *Alternaria* fungi. It naturally occurs on fruits, vegetables and cereals like apples, tomatoes or wheat (Weidenbörner, 2001) and has also been obtained by total synthesis (Koch *et al.*, 2005). The molecular structure of the title compound and the atom-labeling scheme are shown in Fig. 1. It is noteworthy that the benzene rings are not fully coplanar. This phenomenon is not observed for the benzo[c]chromen-6-one analogue 2-chloro-7-hydroxy-8-methyl-6*H*-benzo[c]chromen-6-one (Appel *et al.*, 2006). Hence, the lacking planarity of the alternariol molecule may be attributed to a steric effect caused by the proximity of the H6A hydrogen to the C14 methyl group, which is not present in the planar analogue. This explanation is corroborated by the fact that the C8—C13—C14 angle is increased to 124.93 (17)°. The absolute configuration cannot be derived confidently since the molecule is a weak anomalous scatterer, which is documented by a large s.u. value for the Flack X parameter. Besides the intramolecular hydrogen bonds between O3—H3 and O2 (see dashed blue bonds in fig. 2), each molecule is connected to four adjacent molecules via intermolecular hydrogen bonds (see dashed green bonds in fig. 2). As a result undulated layers in the the ac plane are formed.

### Experimental

Alternariol was supplied by the workgroup of Prof. R. Faust (University of Kassel, Germany) by total synthesis according to a literature procedure (Koch *et al.*, 2005). Alternariol crystals were grown by sublimation in argon atmosphere. To do so, 100 mg of crude alternariol were heated to 380 °C for 2.5 h under a slow argon flow (atmospheric pressure). After cooling to room temperature, colourless needles could be collected from the water cooled compartment of the reaction vessel.

### Refinement

In the absence of anomalous scatterers, the absolute structure cannot be determined therefore Friedel pairs were merged prior to refinement. The hydrogen atoms were located in difference maps and refined with U_iso_(H) set to 1.2 U_eq_ of the parent atom (1.5 for methyl groups).

### Crystal data

**Table:**

C_14_H_10_O_5_	*F*(000) = 536
*M**_r_* = 258.22	*D*_x_ = 1.594 Mg m^−^^3^
Orthorhombic, *P**c**a*2_1_	Mo *K*α radiation, λ = 0.71073 Å
Hall symbol: P 2c -2ac	Cell parameters from 1894 reflections
*a* = 18.969 (3) Å	θ = 5–26°
*b* = 3.7244 (6) Å	µ = 0.12 mm^−^^1^
*c* = 15.235 (3) Å	*T* = 150 K
*V* = 1076.3 (3) Å^3^	Needle, colourless
*Z* = 4	0.40 × 0.10 × 0.02 mm

### Data collection

**Table:**

Stoe IPDS diffractometer	1053 reflections with *I* > 2σ(*I*)
Radiation source: fine-focus sealed tube	*R*_int_ = 0.067
graphite	θ_max_ = 28.1°, θ_min_ = 2.5°
rotation method scans	*h* = −24→22
6072 measured reflections	*k* = −4→4
1338 independent reflections	*l* = −20→20

### Refinement

**Table:**

Refinement on *F*^2^	Primary atom site location: structure-invariant direct methods
Least-squares matrix: full	Secondary atom site location: difference Fourier map
*R*[*F*^2^ > 2σ(*F*^2^)] = 0.035	Hydrogen site location: inferred from neighbouring sites
*wR*(*F*^2^) = 0.069	H atoms treated by a mixture of independent and constrained refinement
*S* = 0.99	*w* = 1/[σ^2^(*F*_o_^2^) + (0.0314*P*)^2^] where *P* = (*F*_o_^2^ + 2*F*_c_^2^)/3
1338 reflections	(Δ/σ)_max_ < 0.001
191 parameters	Δρ_max_ = 0.16 e Å^−^^3^
1 restraint	Δρ_min_ = −0.23 e Å^−^^3^

### Special details

**Table:**

Geometry. All esds (except the esd in the dihedral angle between two l.s. planes) are estimated using the full covariance matrix. The cell esds are taken into account individually in the estimation of esds in distances, angles and torsion angles; correlations between esds in cell parameters are only used when they are defined by crystal symmetry. An approximate (isotropic) treatment of cell esds is used for estimating esds involving l.s. planes.
Refinement. Refinement of *F*^2^ against ALL reflections. The weighted *R*-factor wR and goodness of fit *S* are based on *F*^2^, conventional *R*-factors *R* are based on *F*, with *F* set to zero for negative *F*^2^. The threshold expression of *F*^2^ > σ(*F*^2^) is used only for calculating *R*-factors(gt) etc. and is not relevant to the choice of reflections for refinement. *R*-factors based on *F*^2^ are statistically about twice as large as those based on *F*, and *R*- factors based on ALL data will be even larger.

### Fractional atomic coordinates and isotropic or equivalent isotropic displacement parameters (Å^2^)

**Table:**

	*x*	*y*	*z*	*U*_iso_*/*U*_eq_	
O1	0.54295 (9)	0.3728 (6)	0.78417 (11)	0.0213 (4)	
O2	0.44464 (9)	0.1215 (6)	0.74006 (11)	0.0272 (5)	
O3	0.42666 (9)	−0.0612 (6)	0.57652 (13)	0.0277 (5)	
H3	0.4192 (17)	−0.038 (10)	0.632 (2)	0.033*	
O4	0.61983 (10)	0.1209 (7)	0.38531 (11)	0.0275 (5)	
H4	0.5946 (18)	0.002 (10)	0.344 (2)	0.033*	
O5	0.74389 (10)	0.8553 (6)	0.91670 (10)	0.0260 (5)	
H5	0.7843	0.9307	0.9038	0.031*	
C1	0.50516 (13)	0.2203 (8)	0.71956 (16)	0.0193 (6)	
C2	0.53589 (13)	0.1856 (8)	0.63425 (15)	0.0167 (6)	
C3	0.49365 (13)	0.0493 (8)	0.56447 (16)	0.0192 (6)	
C4	0.52045 (14)	0.0246 (8)	0.48049 (17)	0.0194 (6)	
H4A	0.4917 (15)	−0.072 (8)	0.4295 (19)	0.023*	
C5	0.58935 (13)	0.1357 (9)	0.46651 (15)	0.0183 (6)	
C6	0.63205 (15)	0.2688 (8)	0.53304 (15)	0.0178 (6)	
H6A	0.6788 (15)	0.341 (8)	0.5183 (18)	0.021*	
C7	0.60688 (12)	0.2911 (7)	0.61830 (15)	0.0135 (5)	
C8	0.64850 (13)	0.4228 (7)	0.69286 (14)	0.0141 (5)	
C9	0.61339 (12)	0.4710 (8)	0.77287 (16)	0.0166 (6)	
C10	0.64335 (14)	0.6136 (8)	0.84775 (16)	0.0194 (6)	
H10	0.6189 (14)	0.644 (8)	0.9006 (18)	0.023*	
C11	0.71326 (13)	0.7093 (8)	0.84418 (16)	0.0186 (6)	
C12	0.75199 (13)	0.6479 (8)	0.76803 (16)	0.0159 (5)	
H12	0.7968 (16)	0.696 (8)	0.7685 (17)	0.019*	
C13	0.72187 (13)	0.5072 (7)	0.69300 (15)	0.0146 (5)	
C14	0.77182 (13)	0.4380 (8)	0.61791 (16)	0.0198 (6)	
H14A	0.8205	0.4683	0.6382	0.030*	
H14B	0.7622	0.6083	0.5704	0.030*	
H14C	0.7652	0.1923	0.5964	0.030*	

### Atomic displacement parameters (Å^2^)

**Table:**

	*U*^11^	*U*^22^	*U*^33^	*U*^12^	*U*^13^	*U*^23^
O1	0.0154 (8)	0.0343 (13)	0.0141 (8)	−0.0028 (8)	0.0031 (6)	−0.0013 (9)
O2	0.0164 (9)	0.0475 (14)	0.0177 (8)	−0.0068 (9)	0.0028 (7)	0.0060 (9)
O3	0.0125 (9)	0.0439 (14)	0.0268 (10)	−0.0096 (9)	0.0011 (8)	−0.0021 (11)
O4	0.0164 (9)	0.0518 (15)	0.0143 (9)	−0.0034 (10)	−0.0002 (7)	−0.0092 (9)
O5	0.0244 (10)	0.0405 (13)	0.0131 (8)	−0.0053 (9)	−0.0034 (7)	−0.0063 (9)
C1	0.0152 (13)	0.0243 (18)	0.0183 (13)	−0.0006 (11)	−0.0013 (9)	0.0027 (11)
C2	0.0139 (12)	0.0187 (16)	0.0176 (12)	0.0002 (10)	0.0010 (9)	0.0038 (11)
C3	0.0113 (12)	0.0239 (17)	0.0222 (13)	−0.0027 (11)	−0.0021 (10)	0.0012 (12)
C4	0.0166 (13)	0.0218 (17)	0.0200 (12)	0.0027 (11)	−0.0052 (10)	−0.0022 (11)
C5	0.0149 (13)	0.0277 (17)	0.0123 (11)	0.0025 (12)	0.0021 (9)	−0.0019 (12)
C6	0.0134 (12)	0.0226 (17)	0.0175 (12)	0.0001 (10)	0.0012 (9)	−0.0003 (11)
C7	0.0124 (11)	0.0153 (15)	0.0127 (11)	0.0024 (9)	0.0007 (9)	0.0026 (11)
C8	0.0170 (12)	0.0150 (14)	0.0102 (10)	0.0036 (10)	−0.0006 (9)	−0.0001 (10)
C9	0.0129 (11)	0.0216 (17)	0.0153 (10)	0.0008 (10)	−0.0006 (10)	0.0035 (11)
C10	0.0207 (14)	0.0257 (17)	0.0117 (11)	0.0037 (11)	0.0030 (9)	0.0001 (11)
C11	0.0205 (13)	0.0227 (17)	0.0125 (11)	0.0014 (11)	−0.0061 (10)	−0.0013 (11)
C12	0.0124 (11)	0.0191 (15)	0.0163 (11)	0.0014 (10)	−0.0020 (9)	0.0014 (11)
C13	0.0166 (12)	0.0139 (15)	0.0133 (10)	0.0023 (10)	0.0015 (9)	0.0030 (10)
C14	0.0130 (12)	0.0279 (17)	0.0184 (11)	−0.0009 (10)	0.0004 (9)	−0.0038 (12)

### Geometric parameters (Å, °)

**Table:**

O1—C1	1.343 (3)	C6—C7	1.386 (3)
O1—C9	1.396 (3)	C6—H6A	0.95 (3)
O2—C1	1.245 (3)	C7—C8	1.468 (3)
O3—C3	1.348 (3)	C8—C9	1.401 (3)
O3—H3	0.86 (3)	C8—C13	1.427 (4)
O4—C5	1.367 (3)	C9—C10	1.381 (4)
O4—H4	0.91 (4)	C10—C11	1.374 (4)
O5—C11	1.362 (3)	C10—H10	0.94 (3)
O5—H5	0.8400	C11—C12	1.392 (4)
C1—C2	1.430 (3)	C12—C13	1.381 (4)
C2—C7	1.424 (3)	C12—H12	0.87 (3)
C2—C3	1.425 (4)	C13—C14	1.508 (3)
C3—C4	1.380 (3)	C14—H14A	0.9800
C4—C5	1.387 (4)	C14—H14B	0.9800
C4—H4A	1.01 (3)	C14—H14C	0.9800
C5—C6	1.389 (4)		
			
C1—O1—C9	122.07 (19)	C9—C8—C13	115.7 (2)
C3—O3—H3	105 (2)	C9—C8—C7	117.4 (2)
C5—O4—H4	115 (2)	C13—C8—C7	126.8 (2)
C11—O5—H5	109.5	C10—C9—O1	113.1 (2)
O2—C1—O1	115.6 (2)	C10—C9—C8	124.9 (2)
O2—C1—C2	125.2 (2)	O1—C9—C8	121.9 (2)
O1—C1—C2	119.1 (2)	C11—C10—C9	117.7 (2)
C7—C2—C3	120.2 (2)	C11—C10—H10	118.7 (17)
C7—C2—C1	121.0 (2)	C9—C10—H10	123.6 (17)
C3—C2—C1	118.8 (2)	O5—C11—C10	118.9 (2)
O3—C3—C4	116.9 (2)	O5—C11—C12	121.1 (2)
O3—C3—C2	122.5 (2)	C10—C11—C12	120.0 (2)
C4—C3—C2	120.6 (2)	C13—C12—C11	122.2 (2)
C3—C4—C5	118.0 (2)	C13—C12—H12	119.3 (18)
C3—C4—H4A	122.4 (16)	C11—C12—H12	118.4 (18)
C5—C4—H4A	119.6 (16)	C12—C13—C8	119.2 (2)
O4—C5—C4	121.7 (2)	C12—C13—C14	115.6 (2)
O4—C5—C6	115.4 (2)	C8—C13—C14	125.1 (2)
C4—C5—C6	122.9 (2)	C13—C14—H14A	109.5
C7—C6—C5	120.3 (2)	C13—C14—H14B	109.5
C7—C6—H6A	121.6 (17)	H14A—C14—H14B	109.5
C5—C6—H6A	118.1 (17)	C13—C14—H14C	109.5
C6—C7—C2	118.0 (2)	H14A—C14—H14C	109.5
C6—C7—C8	124.1 (2)	H14B—C14—H14C	109.5
C2—C7—C8	118.0 (2)		
			
C9—O1—C1—O2	174.9 (2)	C6—C7—C8—C9	172.4 (3)
C9—O1—C1—C2	−5.6 (4)	C2—C7—C8—C9	−6.5 (4)
O2—C1—C2—C7	−176.9 (3)	C6—C7—C8—C13	−7.9 (4)
O1—C1—C2—C7	3.6 (4)	C2—C7—C8—C13	173.3 (3)
O2—C1—C2—C3	4.3 (4)	C1—O1—C9—C10	−178.2 (3)
O1—C1—C2—C3	−175.2 (3)	C1—O1—C9—C8	1.3 (4)
C7—C2—C3—O3	178.6 (3)	C13—C8—C9—C10	4.5 (4)
C1—C2—C3—O3	−2.6 (4)	C7—C8—C9—C10	−175.7 (3)
C7—C2—C3—C4	−1.3 (4)	C13—C8—C9—O1	−174.9 (2)
C1—C2—C3—C4	177.5 (3)	C7—C8—C9—O1	4.8 (4)
O3—C3—C4—C5	−179.8 (3)	O1—C9—C10—C11	178.2 (3)
C2—C3—C4—C5	0.1 (4)	C8—C9—C10—C11	−1.3 (4)
C3—C4—C5—O4	−179.9 (3)	C9—C10—C11—O5	179.1 (2)
C3—C4—C5—C6	0.1 (5)	C9—C10—C11—C12	−2.6 (4)
O4—C5—C6—C7	−179.1 (3)	O5—C11—C12—C13	−178.6 (3)
C4—C5—C6—C7	0.9 (5)	C10—C11—C12—C13	3.1 (4)
C5—C6—C7—C2	−2.1 (4)	C11—C12—C13—C8	0.3 (4)
C5—C6—C7—C8	179.1 (3)	C11—C12—C13—C14	−176.7 (3)
C3—C2—C7—C6	2.3 (4)	C9—C8—C13—C12	−3.9 (4)
C1—C2—C7—C6	−176.5 (3)	C7—C8—C13—C12	176.4 (3)
C3—C2—C7—C8	−178.8 (3)	C9—C8—C13—C14	172.9 (3)
C1—C2—C7—C8	2.5 (4)	C7—C8—C13—C14	−6.8 (4)

### Hydrogen-bond geometry (Å, °)

**Table:**

*D*—H···*A*	*D*—H	H···*A*	*D*···*A*	*D*—H···*A*
O3—H3···O2	0.86 (3)	1.82 (3)	2.605 (3)	152 (3)
O4—H4···O2^i^	0.91 (3)	1.81 (3)	2.685 (3)	162 (3)
O5—H5···O4^ii^	0.84	1.97	2.809 (2)	175

Symmetry codes: (i) −*x*+1, −*y*, *z*−1/2; (ii) −*x*+3/2, *y*+1, *z*+1/2.
